# Editorial: Insights in veterinary experimental and diagnostic pathology: 2023

**DOI:** 10.3389/fvets.2024.1437289

**Published:** 2024-06-11

**Authors:** Francisco J. Salguero

**Affiliations:** ^1^Pathology Department, UK Health Security Agency (UKHSA), Porton Down, Salisbury, United Kingdom; ^2^School of Biosciences and Medicine, University of Surrey, Guildford, United Kingdom

**Keywords:** pathology, modeling, organoid, infectious disease, genetics, microRNA

The veterinary pathology discipline is experiencing dramatic changes. The diagnostic techniques used by veterinary pathologists are incorporating new methodologies with a focus on molecular detection, digitalisation and the incorporation of digital analysis and artificial intelligence (1). New technologies are also being used in research pathology, to understand the pathogenesis of disease and to be in line with the 3Rs (2, 3). The present Research Topic is focused on new insights in Veterinary Experimental and Diagnostic Pathology, with three reviews, two original research articles and a case report.

Chen et al. reviewed the optimisation and use of organoid technology in veterinary disease modeling. They described the progress from 2D to 3D culture systems and the incorporation of stem cells into organoids. Organoids provide incredible opportunities to study the pathogenesis of disease, and testing for new therapies. This technology has been more widely applied in human diseases modeling and, although being still in its infancy for veterinary disease, the applications will bloom in the near future.

Hatala et al. described an *in vitro* model of feline idiopathic cystitis, a common cause of lower urinary tract disease in cats. Using primary uroepithelial cells from the urinary bladder mucosa, they established a methodology to study the molecular effects of intermittent stress which can lead to inflammatory responses, oxidative stress and decrease barrier function of the uroepithelium.

Varvil and dos Santos reviewed the literature about the use of microRNA detection and expression as potential tools for diagnosis and prognosis of disease in dogs. They discovered abundant literature about the use of microRNAs in normal processes, non-infectious and noninflammatory conditions, infectious and/or inflammatory conditions and neoplasia. They draw attention about the lack of standardization of microRNA evaluation among studies, something to have present for future studies.

On another review article, Hu et al. described the current detection methods for one of the most important infectious diseases in pigs, African swine fever (ASF). ASF has been causing epizootics in Africa, Asia, and Europe in the past decade, and is spreading quickly to other territories. Having efficient diagnostic techniques is crucial to detect promptly the disease in the farms and in the laboratory. The authors discussed the current methods used to detect the ASF viral genome by PCR or, multiple-polymerase and amplification technologies and the detection of antibodies, what can be used as a supplementary approach to study the infectious Status of individual pigs or farms.

Labens et al. described experimental treatment for would healing of skin in horses, using topical propylene glycol gel, and how this affects the histological architecture in skin biopsies and the microbiome. They showed that topical wound applications may alter the resident microbiota during the healing process, opening the door to microbiota manipulation as potential therapeutic use in future studies.

Finally, Park et al. reported an interesting case of *schistosomus reflexus* in a Holstein dairy cattle fetus in Korea, one of the most common congenital malformations in bovines, analyzing and identifying the genetic mutations associated with this disorder.

## Author contributions

FS: Writing – original draft, Writing – review & editing.
